# Exploring the Perspectives of Older People on the Concept of Home

**DOI:** 10.1155/2019/2679680

**Published:** 2019-06-18

**Authors:** Deborah Hatcher, Esther Chang, Virginia Schmied, Sandra Garrido

**Affiliations:** Western Sydney University, Sydney, NSW, Australia

## Abstract

**Objectives:**

Continuing to live at home is arguably one of the most important challenges older persons face as they age. The aim of this study was to clarify how older adults conceptualise home through age-related lifestyle changes.

**Methods:**

Principles from grounded theory were used to examine the perspectives of 21 older adults obtained from three focus group discussions and 10 in-depth semistructured interviews.

**Results:**

Four major categories were developed: “anchoring self,” “enabling freedom,” “being comfortable,” and “staying in touch.” *Discussion*. For the participants in this study remaining at home enabled a sense of independence and freedom, self-worth and identity, comfort, and an ongoing active role in the community. However, some aspects of home could be renegotiated despite changes to living location, with new social connections able to be forged and personal comforts being transferrable. This holds important implications for supporting older persons to both sustain living at home and to adjust to changing circumstances, suggesting the importance of drawing on the experiences of older persons themselves in developing strategies to promote successful aging.

## 1. Background

Countries around the globe are experiencing an increase in the proportion of their citizens that are aged 60 and over [1]. Economically, an ageing population is associated with decreasing productivity and higher government spending [2, 3]. Part of this increased spending by governments relates to the provision of institutional aged care and other health services [4]. Thus, sustaining living at home for older people has become high on the agenda of governments in countries such as Australia and the UK [5, 6].

Home relates to both the spatial location in which one resides and to social and psychological constructs [7, 8]. One review of 49 articles found that the concept of home encompassed descriptions of the house itself along with discussions about family, the self, gender, the home as a haven, and the idea of journeying [9]. Another study of the meaning of home in palliative care situations from the nursing literature similarly found that the concept involved attributes related to the physical location, as well as to an emotional environment [10]. In the current study, although we focus on defining the concept of home from the perspectives of the older adults who participated in our study, we follow Kontos' definition [11] and use the term “home” in a broad sense to refer to a physical location in which the individual lives, “a space that is controlled by and is uniquely the domain of the individual” (p. 179).

Individuals report a preference for staying in their own homes rather than relocating to care accommodation as they age [12]. In Australia, 99% of people between the ages of 65 and 74 and 75% of people over the age of 85 were living in private dwellings in 2006 [13].

Older people tend to spend up to 72% of their time inside their homes [14], making the decision about where to live a crucial one. Being able to continue living at home assists the older person by providing a familiar environment within which to contend with the challenges and changes to lifestyle that occur due to the ageing process. Home also often remains the one stable entity in the lives of the older person when everything else is changing. Living at home thus assists older people to retain some control over their daily lives and to maintain some independence and autonomy [15].

Given the importance of the home environment to an older adult's capacity to sustain independent living, much research has explored the concept of home in terms of questions relating to the home's usability, or safety [16], or in adapting to the need for relocation. Johnson and Bibbo [17], for example, used a phenomenological approach to examine the meaning of home in a group of eight older adults shortly after their relocation to nursing homes in the Midwest U.S. They reported that autonomy of decision-making about where to live as well as actively changing their attitudes strongly influenced the capacity for the participants to adapt to their new environment.

The concept of home for older adults has also been explored from gerontological, environmental, and psychological viewpoints [18]. Oswald and Wahl [19], for example, took an environmental psychology perspective and focused on concepts related to attachment to place in 126 older persons living at home in Germany. They found five global meaning categories relating to the concept of attachment to home: physical, behavioural, cognitive, emotional, and social.

Another study focused on home as a “space” among widows, showing that emotional attachments to “home” are multifaceted and are strongly influenced by personal connection to place and relationships [20]. Barry and colleagues [21] performed a concept analysis of 49 published articles to define a concept of home from older women living alone in the community. They found that to older women home was a resource and an attachment that took an increasing effort to sustain, but that maintaining one's capacity to stay living at home was a cultural expectation.

In other research, the concept of home has been explored with a view to understand the housing needs of older individuals. Bigonnesse and colleagues [22], for example, found that personal belongings that hold memories and bring comfort connect older persons to the physical space of home. For some, a house itself can be a place of familial heritage and allows the individual to remain connected to the generations of family members who once lived within the walls [23].

Relationships and roles in the community are also an integral part of the meaning of home and can provide security and comfort [24]. A sense of identity in the community can result from friendly neighbours, nearby friends, and family and participation in volunteer roles or special interest groups [20]. Being forced to leave one's home can sever important community connections and remove the intergenerational aspect of community living.

Understanding individual experiences of remaining at home offers important perspectives that can inform ageing policy and practice in residential and community aged care to support ongoing independence in older adults. Furthermore, to understand the issues older people face when considering their housing options, it is vital to explore the feelings and beliefs of older people about the meaning home has for them. However, as conditions relating to ageing at home are experienced in highly individual ways and older persons in the community include individuals with more than 40 years of collective generational experience, it can be difficult to address the phenomenon of sustaining living at home comprehensively and holistically.

Older persons are not a homogeneous group. The way a person adjusts and adapts to ageing is influenced by individual, biological, psychological, and social factors within the economic and political context in which they live, as well as lifestyle, educational, and environmental factors [25]. Thus, the experience of ageing and adapting to the changes with which ageing is associated is highly individual. Despite this, the subjective experience of older persons who remain in their homes during the ageing process has received comparatively little attention in the literature. Few studies have compared the meaning of home between people who are able to remain in the family home and those who must adjust to new locations. Nevertheless, to gain an accurate understanding of the issues older people face when considering their living options, it is vital to explore the perspectives of older persons themselves on the concept of home.

This study, therefore, aims to understand the phenomenon of older people living at home in Australia at a personal level, from the perspective both of those living in their long-term family home and those who have adjusted to newer living conditions in older age. The study aims to clarify how older adults conceptualise home and provide a perspective that can inform policy and practice to support continued living at home or successful transitions to care accommodation in older adults.

## 2. Method

This study used focus groups and interviews conducted with 21 older adults, drawing on principles of grounded theory [26] in its approach to data collection and analysis. Grounded theory is a qualitative method often used to investigate an individual's experience of a phenomenon and was followed in the current study in that we took an inductive approach to exploring the research question, without prior hypotheses [27]. This approach has been widely used across nursing and midwifery disciplines from pregnancy to end of life care and has been successful in gaining knowledge about areas of limited understanding [28–30].

### 2.1. Participants and Recruitment

Both focus groups and interviews were used to collect data, following the model of Lambert and Loiselle [31]. Purposive sampling was used to recruit participants for the focus groups, and theoretical sampling (with the researcher using data previously collected to determine where to next collect data) was used to recruit interview participants, as is common in the practice of grounded theory and will be described in more detail below [32]. Participants were recruited from a local government Seniors Centre in Western Sydney, Australia. Inclusion criteria were as follows: (i) aged 65 or over; (ii) a resident of Western Sydney NSW, Australia, (iii) currently living in their home for at least 12 months before the study, (iv) English speaking, and (v) consented to participate in the study.

Of the 21 participants in total, seven were married, 12 widowed, one never married, and one divorced. Twelve participants lived in a house, six in a unit and the other three in a town house, retirement village, and a duplex. The length of time participants had lived in their current dwelling ranged from 2.5–74 years. Almost all participants had lived in their current homes for at least 10 years, and some had lived there for more than 40 years. The household composition included 12 living alone, five living with a partner, two with their children, and two with a partner and children. According to local census information, demographics were relatively reflective of the population of older people living in the study's geographical area [33].

#### 2.1.1. Focus Groups

Purposive sampling was first used to develop focus groups which provided a broad collective perspective of the topic. Three focus groups were conducted, and participants were invited to participate following their attendance at regular classes at the Seniors Centre, including line dancing, exercise, and indoor bowls classes. Group 1 consisted of three males and seven females, group 2 consisted of six females, and group 3 consisted of four males. Same gender groups such as those in groups 2 and 3 were used to account for gender-based differences in interaction styles [34].

#### 2.1.2. Interviews

Following the three focus groups, interviews were conducted to provide an opportunity for exploration of topics that were difficult to cover in a group setting such as personal finances and health and to explore individual perspectives in more depth. Recruitment for the interviews occurred at the conclusion of each focus group. In total, 17 out of 20 focus group participants accepted the invitation to participate in the individual interviews. Based on the preliminary concepts that emerged from the analysis of focus group data, nine older persons were recruited to be interviewed using theoretical sampling. These nine participants were selected because they reflected a range of personal circumstances and variability in the strategies they used to sustain their capacity to continue living at home. They also had various levels of good health and support, which allowed for a wide exploration of circumstances. One other older person who was unable to attend a focus group also accepted the invitation to be interviewed (*n* = 10). Pseudonyms were used, and personally identifying information was removed from transcriptions to ensure participant confidentiality.

Sampling was determined by the need to recruit a participant who could provide the information necessary to further develop the theory. In the first five interviews, four females over the age of 75 years living alone and one male living with a family member were chosen as data showed these participants had already put in place a number of processes to enable continued living at home. In the second group of five interviews, the older persons selected for interview were chosen because their individual circumstances had changed significantly and become more complex due to deteriorating health. These five participants had either moved out of their home into alternative accommodation or were now living at home with higher levels of support. One female participant was interviewed in each group due to changing circumstances over time.

### 2.2. Data Collection

After obtaining ethics approval from the Western Sydney University Human Research Ethics Committee and meeting with the Senior Centre's coordinator, the researcher attended a forum for centre members to explain details of the study. Potential participants were told that the study related to exploring the concept of ageing and remaining living at home with a positive ageing focus. Participants were given both verbal and written information about the study and were given a chance to ask questions about the study. They were told that they could take the information sheet and consent form away and think about participation. However, all those who volunteered wanted to sign up immediately. Older persons who accepted the invitation to participate were personally contacted via telephone within two days of the study to ascertain that they met the inclusion criteria. All volunteers met the inclusion criteria. Written informed consent was obtained from all participants prior to commencing the focus groups.

Consistent with grounded theory, data collection and analysis proceeded in an iterative fashion that moved backwards and forwards from one level of analysis to another. Data collection commenced with a general approach through focus group discussions. As the study progressed, data collection and analysis became more focused through the individual interviews. The concepts generated from this analysis were then used as the basis for questions for the individual interviews. Data collection ceased when theoretical saturation was reached, i.e., when no additional data could be used to further develop the properties of a category [26].

#### 2.2.1. Focus Groups

Focus groups were conducted in a small quiet room that was free from visual and outside distractions with participants seated in a circular fashion behind tables. Discussions were audio recorded using a digital recorder. Biographical data were collected from each person by means of a Participant Personal Profile form, which collected information about personal characteristics, education, occupation, retirement, income, housing, living arrangement, and support. Participants in each focus group were then welcomed and an introduction about the purpose of the focus group given. A topic guide that had been developed based on the literature was used to prompt the discussions following Krueger and Casey's [35] recommendation (see Appendix A). Concept checking was performed by reading the main points to participants for confirmation both during and after data collection [36].

#### 2.2.2. Interviews

The interviews were conducted using a focused, open-ended, and semistructured interview guide to prompt conversation [37]. This approach enabled the researcher to ensure coverage of essential topics while still allowing the interview to be largely directed by the participant (see Appendix B). Each interview gathered more data on the developing categories, and the questions became more focused as the interviews progressed in order to obtain saturation of the developing categories. Data were collected, coded, and analysed to determine where to next collect data, as described below.

Of the nine participants (one interviewed twice), two chose to be interviewed in their own homes and one in a hostel. Another chose to be interviewed over the telephone at the retirement village. The remaining interviews were conducted at the Seniors Centre. All interviews were audio-recorded using a digital recorder.

### 2.3. Data Analysis

Data collection and analysis informed each other throughout the progress of the study [32]. Open coding was first conducted line by line on printed transcripts using a process that Strauss and Corbin described as microanalysis. Initial codes were *in vivo*, that is, in the words of the older persons. Later in analysis, axial coding was used in which codes were conceptualised and initial *in vivo* codes were further developed into categories by grouping of similar concepts and exploration of relationships between the codes. Constant comparison was used, with all new data coded and compared with previously collected data for similarities and differences. During this process, questions were continually asked of the data, diagrams were constructed and memos were written to assist with theoretical conception. In a third level of analysis, selective coding was used to integrate data categories in formation and refinement of an overarching theory [38]. Guided by the Strauss and Corbin [32] approach, a storyline was written to define and explain the central category and the relationship of all categories.

## 3. Results

The study revealed four major categories in the data that related to the perspectives of home of the older adults in this study: *anchoring self, enabling freedom, being comfortable, and staying in touch*. These categories along with their subcategories will be discussed in more detail below (see Table 1).

### 3.1. Anchoring Self

The determination to remain living at home was evident for all older persons in this study. Home represented their past, present, and future and gave the notion of “anchoring self,” as Jane described: *“But one's life gets locked into an area … your life gets locked up in the things you create.”* Within this concept of anchoring self, four factors appeared to increase the sense of connection that individuals felt with their home: (i) longevity of domicile, (ii) personal investment in the home, (iii) the sense of identity and place in the world that the home instilled, (iv) and the facilitation of self-expression that having one's own home provided.

#### 3.1.1. Longevity of Domicile

Most of the older persons had lived in the same home for a significant time such as John, *“I have lived in the same place for 32 years,”* and Sarah, *“I have lived there for 74 years.”* This longevity of domicile created strong and deep connections, resulting in a reluctance to leave. Sarah described how she felt firmly anchored at home and had “*never ever thought about”* living elsewhere. Rose similarly said:

I couldn't imagine being anywhere else …. I love my home and I lived there when I was married and I've never had another home on my own …. I don't want to leave it and I can't imagine going into a nursing home or a retirement village or anything like that because it's just, I suppose, just home to me.

#### 3.1.2. Personal Investment

The investment of personal and financial resources in obtaining one's home further strengthened this sense of attachment to their home. As Irene stated:

Well, why give up a home when I've fought so hard to get it? I had to pay for the place myself …. I have lived in the house for 40 years and I don't want to shift … I've got no intentions unless I have to of leaving. Maybe I'm too determined. I think I'd die before leaving.

Others described how a part of them had gone into building and creating their home. As Peggy said: “*We built our house.”* Kathy further spoke about how she built a new home to facilitate self-management of her chronic illness, taking responsibility for creating her own living circumstances as her needs changed.

#### 3.1.3. Sense of Identity and Place in the World

For many, a major reason that home provided a sense of anchorage for the self related to self-identity. This concept was illustrated by the way older persons consistently made reference to their home as *my home*, implying a sense of identity through ownership. A sense of the status and a place in the world that is achieved by home ownership was also described. Sally said: “*It is my home because I own it.”*

Living in one's home over a significant period of time also facilitated close connections with the local community, which further strengthened this sense of self-identity as being attached to the location of the physical place of residence. Living at home enabled the older persons to maintain this sense of identity within their community as it conveyed messages about them to others. These community connections became even more evident when significant life changes occurred, such as the death of a spouse. Jane described this when she said: *“Those people [neighbours] had known him [her husband] and that is very important to me, that I am not just me but that I was part of a double, part of somebody else.”*

#### 3.1.4. Facilitation of Self-Expression

For others, creating a home was as simple as keeping mementos around the home as a reflection and expression of self. This aspect was illustrated when Ellen made reference to moving from house to house throughout her adult years and creating a new home through decorating each new house with personal mementos. She expressed this as: “*I can make a home anywhere … Everybody has a few personal things that they dearly love* … *I put up two paintings. That was home.”*

In summary, to the older persons in this study, home represented a place where they felt anchored and where they were able to create and maintain their self-identity despite the changes and challenges that occur with ageing. This sense of connection with their home made it difficult for many of these older people to envisage living in alternative accommodation. Home was presented as an extension of the person and extraction from their entrenched lives at home would mean leaving behind a part of themselves, losing their sense of self.

### 3.2. Enabling Freedom

The meaning of home also encompassed being free of constraints. To many participants, home symbolised freedom as highlighted by Dan, *“Because I am sure that living in my home, definitely, [means] more freedom.”* This freedom included (i) the ability to personalize activities and self-manage time, (ii) having a purpose and reasons to keep busy, (iii) performing the tasks of everyday living independently, and (iv) not having to contend with interference from others.

#### 3.2.1. Personalize Activities and Self-Manage Time

Home offered the older persons a known territory and a familiar environment in which they retained self-determination in deciding how they would go about their everyday living. Thus, living at home afforded people the freedom to personalize their daily activities and self-manage their time. Rose expressed this freedom saying: *“Because it is home and I can do what I like.”* Irene similarly identified the control she maintains: *“Come and go when you wish … without someone wanting you to do this or that.”*

#### 3.2.2. Purpose and Reason to Keep Busy

Living independently in one's own home necessitates a certain amount of work in order to care for oneself and the home. Thus, living at home gave individuals a degree of purpose in life and a reason to keep busy. Irene expressed this as: “*I feed my cat and look after that, keep the house tidy in general and I see that the raking up's done out in the garden.”* Sarah said: “*I do my own cooking and things like that … I do all my housework myself.”* The participants recognised that keeping busy and active would help them to maintain good health.

#### 3.2.3. Independence in Everyday Tasks

Although the participants varied in their ability to perform the tasks of everyday living themselves, a range of benefits ensued from being independent, including a sense of self-pride and personal satisfaction through feelings of accomplishment. Many expressed pride at their ability to live independently. Anne and Rose both proudly stated, “*I do all my own work.”* Home gave them the freedom to do this, which might not be possible if they relocated.

#### 3.2.4. Lack of Interference from Others

Independent living enabled the freedom of not having to consider anyone else or contend with interference from others. This freedom facilitated autonomy and control in their planning of everyday living. Rose described her experience as:

I'm used to being on my own and when you're on your own you can do what you like, you can eat what you like, you can go to bed and get up when you feel like it …. I like to be able to do things my way. If you've got me living with anyone they say well you do so and so and they might do it different to what I would do it … I just feel happy living in my own home …. Doing what I want to do and doing it when I feel like doing it.

It was felt that the sense of freedom and independence gained from living at home would be lost with moving out of home. Irene revealed this concern when she said, *“I'm too attached to my home … I'm used to being independent and I don't think I could confine down to regulations and rules.”* Tim, who was interviewed both before and after moving to a hostel commented on the loss to his freedom and independence after the move, saying:

They have fixed meal times here, so you can't wander down any old time… On your own you might say, “Oh, I'll have something early,” or “I'll have it later” … I'm used to sort of going out to the shops and doing a little bit of shopping and now that's a problem.

In summary, home as a place for freedom was one of the most important meanings of home emerging from the data. Older persons described having a choice in making decisions about everyday activities, as well as the capacity to live independently and to keep busy with the tasks of everyday living. This not only resulted in great pride and personal satisfaction but also enabled them to live without the interference of others.

### 3.3. Being Comfortable

A third subcategory emerging from the responses of the older persons as they described the meaning of home was that home was a source of emotional and physical comfort. Comfort was derived from (i) having things one needs, (ii) the good memories associated with home, (iii) the familiar territory providing peace of mind and stability, and (iv) having a space for relaxation and restoration.

#### 3.3.1. Having Things One Needs

Ellen spoke of the contentment derived from living at home when she said, *“It's very comfortable. We're here together … Our home is very important to us … I think we've got everything we want in this house.”* Marjorie echoed Ellen's comments when she said, *“I've got everything there that I want.”* Tim similarly stated,

I had all my stuff there, and it's organised comfortably with comfortable chairs and a good bed and everything… you've got everything, when I'd arranged everything the way I wanted.

Interestingly, “comfort” was one of the meanings of home that older persons were able to renegotiate after relocation. Although only a few older persons had moved out of their homes by the conclusion of the study, all had managed to find comfort through having familiar things around them. After relocating to a hostel, Tim was able to state, “*This is my home … I'm comfortable here.”* Referring to her relocation to a retirement village, Maree similarly said: “*It's very comfortable… happy to be here and it is, with all my things around me, it's just like my own home.”*

#### 3.3.2. Good Memories

Older persons also found comfort from the many good memories their home generated. Home was a place where they could reflect on happy times. Dan spoke of these pleasant memories when he said, *“You cannot deny at our age, our memories, still there is a lot [of memories at] home … and everyone enjoys to see or to live or to tell about his memories.”* Some described home as a place where memories of their childhood, adulthood, and parenthood are held and embodied. Irene stated, “*I have a lot of memories … when no one's here … I have a lot of thinking to do.”* Sarah also described feeling comfort through reliving fond childhood memories of the home where she still resides.

A few older persons also revealed that living at home gave comfort to their adult children who now resided in their own homes. Rose, for example, described the happy memories her home continues to give them. She stated that home was “*your comfort and your memories… memories of the children when they were small… When they* [children] *all married and had their own homes, they call my place home.”*

#### 3.3.3. Familiarity, Peace of Mind, and Stability

The idea of familiarity also seems to underpin the concept of comfort in the older persons in this study. Having comfort at home was also expressed as having peace of mind and a sense of competence through living in a familiar environment. Ellen illustrated this when she said, “*I know where things are so that's a big plus … It's familiar.”* Rose similarly said, “*I'm comfortable … I know where everything is that I need … even if I didn't have my fairly good eyesight I would still be able to get around.”* This familiarity evidently added to a sense of happiness and wellbeing in participants. As Peggy stated, *“I have been there [home] for about 50 years now and I enjoy it.”*

#### 3.3.4. Space for Relaxation and Restoration

Living at home and having access to their own garden also provided participants with a comfortable place for relaxation. Sally said, *“It's a sort of relaxation to go down there [garden]*.” For some, their garden had become a haven to unwind and a place to take things easy. Irene described her experience as: *“You forget your worries I reckon when you go outside.”* She also stated, “*I like space … would hate to live in a room. I like my backyard.”*

Being able to use home as a place for revival and restoration when returning home after engaging with others in the community was also a way of being comfortable. Ellen described being at home between activities and social outings as*: “You stay at home to catch your breath.”*

In summary, being comfortable was an important subcategory describing the meaning of home emerging from the responses of older persons in this study. Living at home provided emotional and physical comfort as home afforded the physical comforts of having the things one needs organized as one pleased, connection with good memories, a peaceful space for relaxation and restoration, and peace of mind and stability through familiarity.

### 3.4. Staying in Touch

The final subcategory which emerged from the data was “staying in touch.” The older persons agreed that living at home (i) positioned them close to their family, friends, and neighbours and (ii) provided a base for participation in community activities and making new friends.

#### 3.4.1. Positioning Close to Family and Friends

The consensus among the older persons in this study was that living at home positioned them close to their family, friends, and neighbours. In this way, home provided a support network and prevented isolation, allowing them to keep in contact with others. When describing her available support network Sarah stated, *“My sister lives next door,”* and Ellen referred to her support as: *“We have got our family around there. We got our interests all round this area …. It's just in a good position …. We've got a daughter down at (−).”*

The older persons in this study all agreed that living at home enabled them to maintain ties with people with whom they had longstanding relationships. Living at home was seen to facilitate staying in touch with others, as home and its community is known territory, and people get to know each other over time. Marjorie explained the meaning attached to these relationships with others over time when she said:

The people around here, the neighbours around me, we've all been together there … since 1950 … The majority of them are the same but we've got some new neighbours and they're up the top end of the street and you wouldn't even know their name … but the ones down where we are, there's only about six or eight houses, they all pitch in and help one another.

#### 3.4.2. A Base for Community Participation

As home was known territory, it provided a base, a way to get out into the wider community and return. When using home as a base to go out and participate in activities in the community, home also became a link to making friends. The importance of this characteristic was reinforced by Maree when she said, “*As you get older it is harder to make new friends.”*

For those who were still living in their long-term home, they saw their established relationships as important to their wellbeing and were reluctant to move away for fear of losing them. However, those who had moved to new homes had made efforts to stay socially connected by establishing new social contacts.

Much of the way living at home facilitated community participation had to do with the familiarity and confidence participants expressed in local options for transport and mobility. Mary and Kathy described how they were able to stay in touch with their communities as their homes enabled access to familiar services which provided them with daily options. Kathy said: “*I like this area because I can go on my $1 ticket everywhere and there is so much to do.”* Sally described her home as enabling her to stay in touch with others because, “*it is convenient to everything.”* The location of their home to enable convenience to facilities and services close to their home or located within the local community, including interests, activities, shops, and their GP, was noted as important. Alice described having the accessibility of these services from her home enabled her to stay in touch when she said, *“It is very convenient where I live to the shops, the buses and the trains.”* Sarah similarly commented, *“It is very convenient where I live, the shops, a doctor and a chemist and other supermarkets.”*

In summary, one of the four meanings of home formed from the responses of the older persons in this study was that living at home enabled them to stay in touch with their community. Through living at home, participants were able to maintain stable relationships and remain socially connected to familiar support networks and services. Living in a familiar environment promoted an awareness of access to transport and other facilities, which provided a means to get out into the community to engage in social or other activities.

## 4. Discussion

This study explored the experiences and perspectives of older people living at home in Western Sydney, Australia. Drawing on principles of grounded theory, focus groups and in-depth interviews were conducted with 21 different participants about the concept of home and what it means to them. The study was able to gain the perspective both of those who were living in their long-term family home, and those who had adjusted to newer living environments in later life.

The data revealed that for the older persons in this study, home was the centre of their everyday living and was critical to maintaining independence, comfort, and freedom. Home was pivotal to the older persons self-identity and as such reflected their sense of self. Continued living in a place to which they had long-term connections and had invested considerable resources to attain, added to a sense of being “anchored” to their living environment and having a place in the world. Living in one's own home also enabled individuals a sense of self-expression in that they were able to style and decorate their homes in their own personal way. This is similar to the findings of Cristoforetti and colleagues [20] who highlighted the importance of showcasing belongings in the home as an extension of the self. Other research has similarly shown the relevance of objects of sentimental value in maintaining a connection with memories of family, friends, and life events [18, 24].

The concept of home was also closely associated with having a sense of freedom for the older people in this study. They described the sense of pride and competence that being able to remain independent gave them, as well as the satisfaction of being able to self-manage their time and organize their days to suit themselves without outside interference. These findings are supported by Sixsmith and colleagues [39] who similarly found that everyday life at home enabled adults over the age of 75 to continue to be engaged in purposeful meaningful action.

In addition, the older persons in this study expressed that the concept of home represented comfort for them in that they were able to have the things they needed for daily comfort around them arranged in ways that suited them. They also derived emotional comfort from the familiarity and stability of remaining in their own home as well as from the connection to happy memories that being in the home gave them. In addition, being at home enabled them to access the outdoors and to have greater space than they would have in institutionalized living arrangements.

Furthermore, participants in the current study reported that home was associated with the capacity to stay in touch with family and friends as well as local services and activities. Living at home facilitated this ongoing connection both by its physical proximity to people with whom the older persons had long-term relationships and by its familiarity, which enhanced confidence in being able to access transport and local services. Bigonesse and colleagues [22] similarly found that social contacts among neighbours reinforced social ties among older adults and helped them to feel socially supported. It may be that this aspect of familiarity with the local environment is particularly of importance as the driving capacity of older adults diminishes, reducing their capacity to engage with the local community. Studies have found that driving cessation is associated with almost doubled risk of depression in older adults [40].

Overall, the current study supported Oswald and Wahls' [19] framework in which they argued that concepts of home fell into five broad meaning categories: physical, behavioural, cognitive, emotional, and social. “Anchoring self” in the current study largely related to emotional and cognitive aspects of the meaning of home, in that it was closely connected with self-identity and a sense of stability. The second meaning in the current study, “Freedom” related to behavioural and cognitive aspects in that it concerned the ability of individuals to stay independent and busy, although this was also associated with emotional aspects in that it facilitated a sense of pride and self-competence. Similarly, “Comfort” also described the emotional comfort in that living at home provided the opportunity to be surrounded by happy memories. This aspect, however, also related strongly to the physical comforts of home. “Staying in touch” concerned both social and behavioural aspects, in that remaining at home enabled people to stay connected with family and friends through physical proximity while also maintaining confidence in the ability to negotiate mobility. Thus, concepts of home in older adults are multifaceted, strongly connected to the physical location and surroundings, but having a deep impact on both emotions and the activities of daily life. These findings have relevance to policy development both for supporting those who remain living at home and to those who choose to relocate to care accommodation.

Nevertheless, it is not always possible or even advantageous for all older adults to remain living at home. For example, some older adults living alone can be at risk of social isolation and difficulty coping with the physical demands of caring for one's self [41]. Individuals with dementia who are living at home can also experience social disconnection and a lack of professional care to cope with psychological symptoms [42]. Despite the appeal to many of continuing to live in their own homes and “ageing in place,” the current study was also able to demonstrate that some aspects of home can be renegotiated despite a change in physical location. Participants who had relocated from their long-term home had been able to successfully adjust, reporting that their new environment now felt like home. They seemed able to do this because of the ability to retain some of the comforts of home and personal possessions and the capacity to make new social connections, despite having to make some concessions to change. However, the ability to renegotiate a new environment may be highly dependent upon the flexibility of the systems present in the new living environment in addition to the older person's ability to adjust.

The current study is limited by its geographical focus on Western Sydney in Australia, the perspectives of which may not always be applicable to other cultural groups or socioeconomic demographics. Furthermore, participants in the current study may have been biased towards considering the concept of home from a positive perspective both by cultural rhetoric and by the way questions were framed. Since the participants in this study were relatively active, having been recruited from a sample who had been participating in physical activity sessions at the Seniors Centre, it should also be noted that the perspectives of home discussed in this article may not represent the points of view of less active older persons.

Nevertheless, this study thus has important implications for the development of policy and practice around both supporting healthy ageing and in facilitating adjustment to new environments where unavoidable. By furthering the understanding of what home means to older adults, strategies can be developed for reinforcing the essential meaning components of home throughout the changes associated with ageing, ensuring that programs are in place to support ongoing independence, self-management, and social connection. Similarly, having a broad knowledge of the meaning of home to older adults can inform the implementation of programs to smooth transitions to new living environments in older adults by reinforcing elements of care that support independence and personalisation. Future research could also benefit from looking more closely at the strategies and personal qualities that older people draw on in both sustaining living at home and in adjusting to changed living circumstances.

## Figures and Tables

**Table 1:** The meaning of home: categories and subcategories.

Anchoring self	Enabling freedom	Being comfortable	Staying in touch
Longevity of domicile	Personalize activities and self-manage time	Having things one needs	Position close to family and friends
Personal investment	Purpose and reason to keep busy	Good memories	Base for participation in community activities
Sense of identity/place in the world	Independence in everyday tasks	Familiarity, peace of mind and stability	
Facilitating self-expression	Lack of interference from others	Space for relaxation and restoration	

## Data Availability

Permission was not sought from participants at the time of consent to archive data, so this is not publicly available.

## Appendix

### A. Topic Guide

#### Participant Introduction and Icebreaker Question

- “Introduce yourself and state where you live and why you like living there.”

#### Opening Question

- “Tell me about the things that make it possible for you to remain living in your home in the community?”

#### Areas to Cover

- Home
- Community
- Independence
- Health and wellbeing
- Resources
- Social network
- Support

### B. Interview Guide

#### Interviews 1–5

- What are the most rewarding aspects of living in your home?
- In what ways does your health enable you to live at home?
- What do you think are your personal attributes (qualities) that enable you to stay living at home?

#### Interviews 6–10

- What is it about your home that makes you want to stay living there?
- What are the things that have helped you to stay living there?
- What are the things that sometimes make it difficult for you to live there?
- What are some of the negative things about living at home for you?
- What are some of the positive things about living at home for you?
